# Complicated Open Wound Management in a Free Clinic Setting

**DOI:** 10.7759/cureus.26605

**Published:** 2022-07-06

**Authors:** Nicholas Blackmond, Emily Provencher, Sarah Provencher, Marim Zoma, Benjamin D Goodman, Alan Silverman

**Affiliations:** 1 Internal Medicine, Dr. Gary Burnstein Community Health Clinic, Pontiac, USA; 2 Internal Medicine, Oakland University William Beaumont School of Medicine, Rochester Hills, USA; 3 Internal Medicine, Loyola University Chicago Stritch School of Medicine, Maywood, USA; 4 Internal Medicine, Henry Ford Health System, Detroit, USA; 5 Cardiology, Henry Ford Health System, Detroit, USA; 6 Medical Education, Oakland University William Beaumont School of Medicine, Rochester Hills, USA

**Keywords:** methicillin resistant staphylococcus aureus (mrsa), wound complication, type-2 diabetes mellitus, free clinics, abscess management

## Abstract

Wound healing is a complex and integrated process that involves several interdependent overlapping stages, including hemostasis, inflammation, proliferation, and vascularization. Cellulitis and skin abscesses are among the most common skin and soft tissue infections. Cellulitis typically involves the deeper dermis of subcutaneous fat and tends to have a more indolent course with the development of localized symptoms over a few days. Skin abscesses are described as a collection of pus within the dermis or subcutaneous space. Diabetes mellitus (DM) is the leading cause of impaired wound healing and consequently has higher rates of patients developing soft tissue infections. Diabetic patients experience decreased early inflammatory cell infiltration but increased numbers of neutrophils and macrophages. Complications include bacteremia, metastatic infection, sepsis, and toxic shock syndrome.

In this case, we describe a 50-year-old Caucasian uninsured male who was referred to the Gary Burnstein Clinic (GBC) from a nearby hospital for wound management after an incision and drainage of a large back abscess and uncontrolled type 2 diabetes mellitus (T2DM). The patient presented with a large erythematous, indurated lesion with a cruciate incision that spanned from his mid-thoracic spine to the medial border of his left scapula. The wound management course required strict follow-up to the clinic every 48-72 hours for debridement and monitoring. This was complicated by the GBC’s limited resources along with the volunteer nurses’ and physicians’ availability. To avoid the patient being lost to follow-up, shared decision-making was utilized to create a schedule that was advantageous for both the patient and the clinic. Ultimately, the patient made a full recovery without any adverse events.

This case highlights the gaps in care for the medically uninsured. We also showcase the passion and dedication our medical volunteers exhibit to care for the community. The GBC provides high-quality healthcare to bridge gaps in access to care by offering broad specialist access while ensuring continuity of care.

## Introduction

Cellulitis, often caused by the bacteria *Staphylococcus aureus*, is one of the most common infections for adult patients seen by primary care physicians, emergency departments, and urgent care. Fluctuant and draining abscesses often accompany cellulitis. Furthermore, cellulitis is the cause of more than 600,000 hospitalizations, and cellulitis with abscesses results in more than 9 million physician office visits per year [1].

Patients with diabetes mellitus (DM) are at higher risk of developing cellulitis. The risk of cellulitis infection is 1.21 times higher in diabetics compared to non-diabetics. Diabetics with glycated hemoglobin (HbA1c) greater than 7.5% have a 40% increased risk of developing cellulitis, and there is a 40% increased risk of developing cellulitis for every 1% increase in HbA1c levels [2]. The challenges of wound management are increased in patients with DM and further complicated in uninsured patients.

Vulnerable populations include people of color, people of lower economic backgrounds, people with low educational backgrounds, and people who are marginalized due to their sexual preference, immigration status, and religion [3]. Due to the high cost of health insurance, patients who are in these demographics often lack health insurance, which can lead to them facing a myriad of obstacles when it comes to social, economic, and healthcare resources. Uninsured patients’ experiences in a healthcare setting are often limited to emergency departments and/or free clinics. This results in these patients having little familiarity with continuity of care in terms of coordination and communication with their healthcare team [3-4]. Individuals lacking health insurance also have increased difficulty obtaining care in comparison to insured patients. This leads to a lack of routine medical visits with a primary care physician; subsequently resulting in unmet health-related needs, a decline in health, and increased rates of hospitalizations. The inability to afford the cost of the multiple and frequent follow-up visits required for wound management can become a barrier for uninsured patients in receiving chronic wound care [3].

Approximately 5 million chronically ill patients in the United States have wounds and the aggregate cost of their care is roughly $20 billion annually [5]. According to the National Hardship and Debt Relief program, patients who are uninsured report more problems accessing care and avoid seeking non-urgent care visits. This could lead to late disease recognition, poor health status, and an increased likelihood of death during hospitalizations [3,6-7]. Additionally, uninsured patients are reportedly less likely to receive any recommended follow-up care from a recent medical visit [6-7].

Social determinants of health are described by the World Health Organization as “non-medical factors that influence health outcomes.” Some of the determinants that influence health equity include income and social protection, education, food insecurity, social inclusion and non-discrimination, and structural conflict [7]. One of the social determinants of health that free clinics can satisfy is quality healthcare services. Yet, free clinics are perceived as providing poor quality care due to various factors such as poor continuity of care and little or no specialist access. Uninsured or low-income patients are more likely to avoid seeking care due to the anxiety and distress of the financial burden that multiple follow-up visits at hospital-affiliated wound care would cause [4,7].

Our patient described in this case was left with little to no options for wound care. From this, we can see where free clinics can bridge the gap by providing high-quality and broad specialist access while ensuring continuity of care [7]. Free clinics also allow patients to overcome financial barriers and out-of-pocket costs associated with care.

## Case presentation

This is a case of a 50-year-old Caucasian uninsured male who was referred to the Gary Burnstein Clinic (GBC) from a nearby hospital for wound management of a large back abscess and newly diagnosed uncontrolled type 2 diabetes mellitus (T2DM). The patient had no significant past medical history and had not seen a physician in 10 years. The patient initially presented to a local emergency department with a two-week history of a boil on his back. He reported trying to express the boil at home and noted that it contained purulent discharge. However, the boil gradually grew to a large erythematous abscess to the medial and lateral border of the left scapula between the superior and inferior aspects of the fourth and seventh thoracic vertebrae, respectively. Incidentally, while the patient was in the emergency department, he was found to have an elevated blood glucose of 405 mg/dL and HbA1c of 12.2%. He was treated with subcutaneous insulin and admitted to surgery for incision and drainage secondary to a diagnosis of cellulitis with a methicillin-susceptible Staphylococcus aureus abscess and started on intravenous cefazolin 2 grams/20 mL for five days and vancomycin 15 mg/kg intravenously. He was discharged in a stable condition with a prescription of amoxicillin/clavulanic acid (Augmentin) 875-125 mg twice a day for five days and a referral to GBC for wound care.

When the patient first arrived at GBC, we were unsure how we were going to be able to meet the patient’s needs. Not only was the abscess larger than anything we had treated, but proper care required him to present every 48-72 hours for debridement and monitoring. Given his new diagnosis of T2DM, we knew this could take months to fully heal, requiring nurse and physician hours outside of the days our clinic typically runs. This was further complicated by all our medical staff being volunteers, many of whom have their own clinics to run or other daily obligations. The alternative was for the patient to follow up with the hospital-affiliated wound care clinic but that would have required the patient to pay out of pocket, which he informed us that he could not afford. Additionally, the patient was reluctant about how often he needed to return to GBC because of the time he had to take off work and subsequently lost wages. However, through shared decision-making with the patient and his dedicated volunteer healthcare team, we successfully created a schedule that was feasible for both the patient and the clinic volunteers as seen in Table 1. This avoided the patient being lost to follow-up.

**Table 1:** Debridement schedule Boxes with 'X' indicate debridement or re-evaluation

November	Monday	Tuesday	Wednesday	Thursday	Friday
11/1 – 11/5					1^st^ visit
11/8 – 11/12	X		X		X
11/15 – 11/19	X				X
11/22 – 11/26	X		X		X
December	Monday	Tuesday	Wednesday	Thursday	Friday
11/29 – 12/3	X		X		X
12/6 – 12/10			X		X
12/13 – 12/17	X		X		X
12/20 – 12/24	X		X		X
12/27 – 12/31	X		X		

At the patient's first visit with the GBC, he still had elevated glycated hemoglobin (HbA1c) at 11.1% and a random blood glucose level of 120 mg/dL. He was managed with insulin therapy, which he was compliant with throughout his time with us. This brought his HbA1c down to 6.4% 60 days after his initial visit shown in Table 2. The patient was found to have iron deficiency anemia during routine blood work, which we attributed to anemia of chronic disease shown in Table 3.

**Table 2:** Trending lab values HbA1c: hemoglobin a1c/glycated hemoglobin; BUN: blood urea nitrogen; eGFR: estimated glomerular filtration rate; AST/SGOT: aspartate aminotransferase/serum glutamic-oxaloacetic transaminase; ALT/SGPT: alanine aminotransferase/serum glutamic-pyruvic transaminase; MCV: mean corpuscular volume; RDW: red cell distribution width

Lab component	Hospital admission	60 days post-admission	Reference
White blood cell	10.0	5.9	3.7 – 11.0 K/UL
Red blood cell	3.0	4.28	4.0 – 6.0 M/UL
Hemoglobin	10.4	11.9	13.0 – 18.0 gm/dL
Hematocrit	26.1	39.1	39.0 – 50.0%
MCV	87	91	80 – 99 fL
RDW	13.4	15.2	12.0 – 15.0%
Platelets	437	327	140 – 440 K/uL
HbA1c	12.2%	6.4%	4.0 – 5.7%
Blood glucose	405	126	70 – 99 mg/dL
Potassium	4.1	4.9	3.5 – 5.3 mEq/L
BUN	6	19	7 – 25 mg/dL
Creatinine	0.50	0.62	0.70 – 1.30 mg/dL
Albumin	2.7	3.7	2.5 – 5.7 g/dL
Bilirubin	0.3	0.5	0.3 – 1.0 mg/dL
eGFR	176	137	>60 mL/min
AST/SGOT	23	18	13 – 39 U/L
ALT/SGPT	22	20	7 – 52 U/L
Alkaline phosphatase	256	100	27 – 120 U/L

**Table 3:** Iron panel TIBC: total iron-binding capacity

Lab component	Values	References
Ferritin	261	38 – 380 ng/mL
Transferrin	223	203 – 362 mg/dL
Iron	49	50 – 212 mcg/dL
TIBC	312	250 – 450 mcg/dL
Iron Saturation	16	20 – 55%

**Figure 1:**
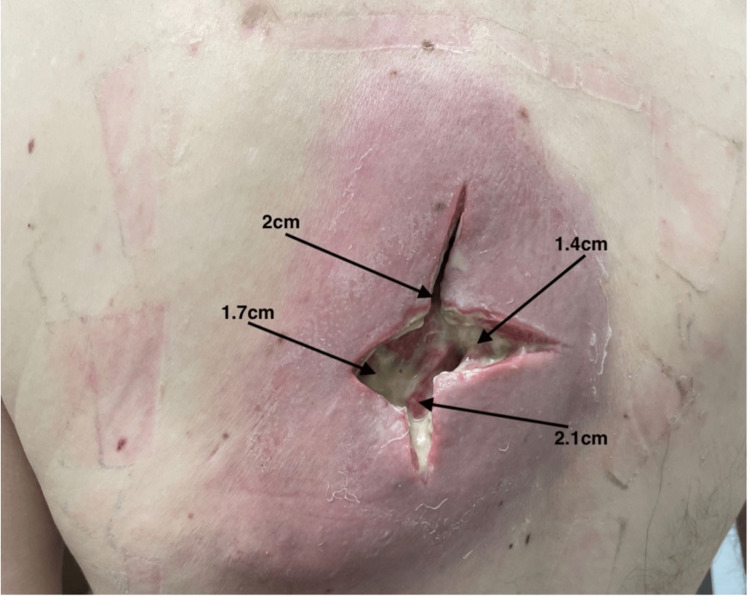
Seven days following hospital admission; first debridement at the Gary Burnstein Clinic 2cm depth at the proximal 12 o'clock position, 1.4cm depth at 3 o'clock position, 2.1cm depth at 6 o'clock position, and 1.7cm depth at 9 o'clock position.

The physical exam showed a large 14 cm x 12 cm, indurated lesion with a cruciate incision. The surrounding tissue was erythematous with purulent drainage. The abscess was located from the superior aspect of the fourth thoracic vertebrae to the inferior aspect of the seventh thoracic vertebrae between the medial and lateral border of the left scapula. The depth of the wound was between 1.2 cm - 1.6 cm shown in Figure 1.

**Figure 2:**
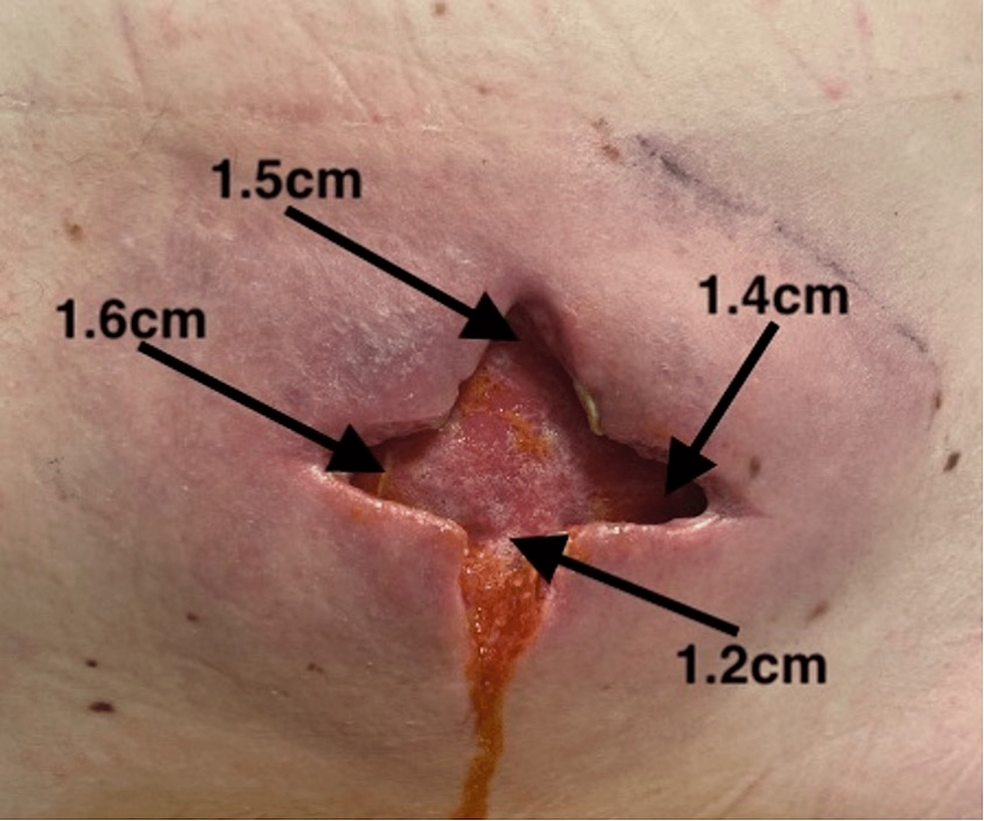
Eleven days following hospital admission; fifth debridement at the Gary Burnstein Clinic 1.5 cm depth at the 12 o'clock position, 1.4 cm depth at the 3 o'clock position, 1.2 cm depth at the 6 o'clock position, and 1.6 cm depth at the 9 o'clock position

**Figure 3:**
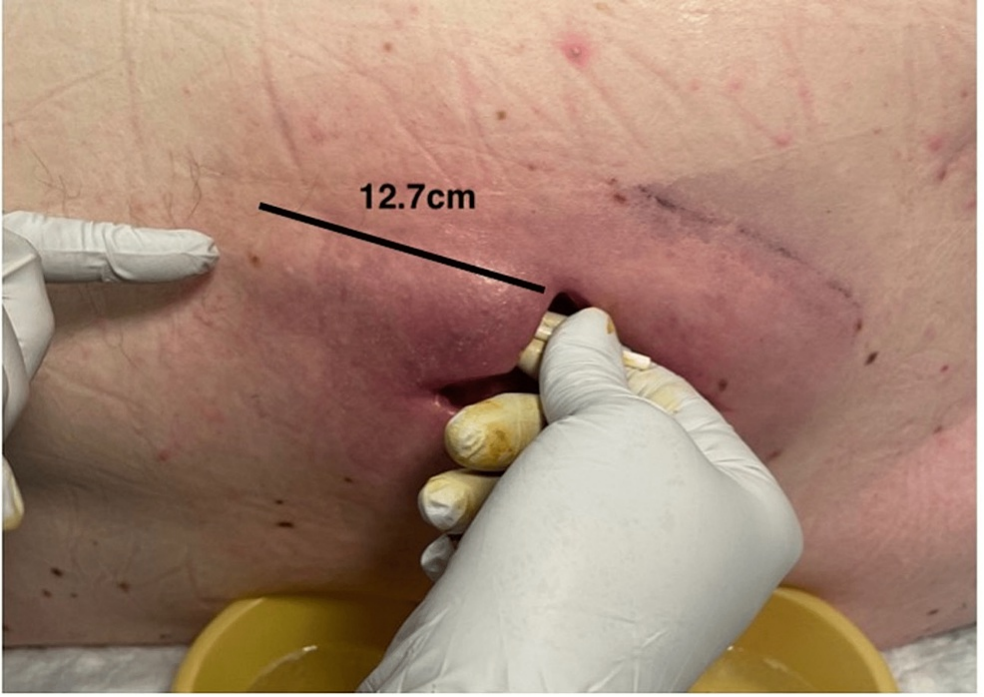
Eleven days following hospital admission; fifth debridement at the Gary Burnstein Clinic 12.7 cm internal cavity length

**Figure 4:**
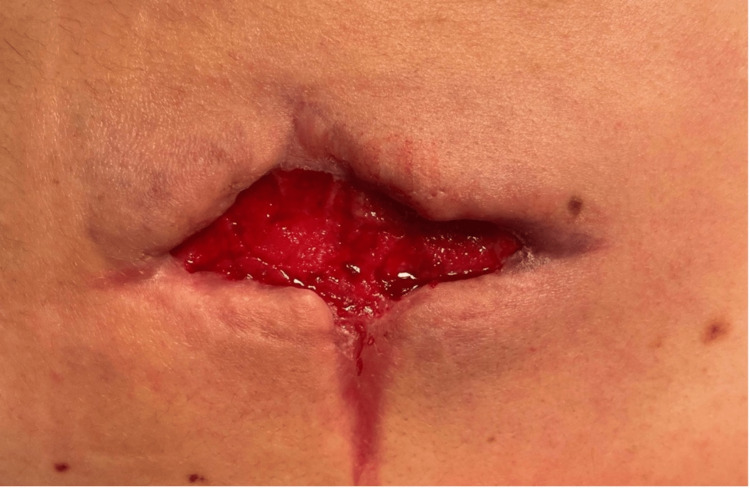
Thirty-seven days following hospital admission; thirteenth debridement at the Gary Burnstein Clinic Minimal surrounding erythema and purulent drainage

**Figure 5:**
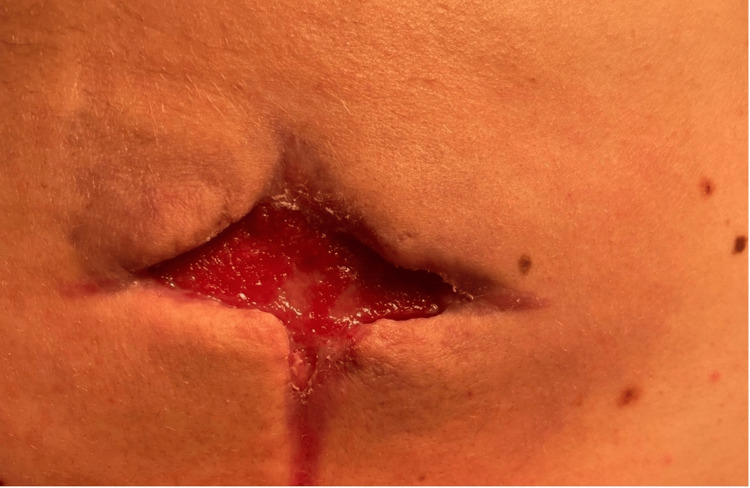
Fifty-three days following hospital admission; twenty-second and final debridement at the Gary Burnstein Clinic Final debridement; edges were approximated and starting to scab

The patient was to follow up every 48-72 hours for debridement for the next two months. By the fourth debridement, the overall depth of the wound showed improvement shown in Figure 2. There was an internal cavity that extended approximately 12.7 cm from the center point to the 3 o’clock position and 9 o’clock position seen in Figure 3. During each visit, the wound was washed out with a 50/50 mixture of Dakins^TM^ solution and sterile water. After washing out the wound, it was packed with sterile Kerlix^TM^ and covered by a 4x4 inch sterile gauze, and secured with a highly absorbable multi-level dressing pad. Each subsequent visit saw more granulation tissue continue to fill the wound while the wound continued to appear healthy and without any signs of infection. Minimal surrounding erythema circumferentially and minimal purulent drainage were also noted in Figure 4. By the final debridement, the wound required minimal attention. The cruciate edges were approximated, they were beginning to scab over, and there were no signs of infection shown in Figure 5.

The patient made satisfactory progress throughout his strict wound care course. When the patient was discharged from the hospital, he was given a five-day course of Augmentin 875-125 mg to be taken twice a day. He did not require additional antibiotics following his debridement care. His T2DM was controlled and doing well. The patient was approved for insurance shortly after his final debridement.

## Discussion

Since the 1970s, there has been a dramatic increase in uninsured and underinsured people seeking free clinics [3,8]. In 1980, the number of people without insurance was near 30 million and has since increased by 50%. This number now hovers around 45 million. Free clinics constitute a large part of the current health care safety net with over 1,000 identified in the US each with an average of over 4,000 patient visits per year and nearly 800 new patients per year [3,8].

Free clinics provide primary care to a substantial number of the uninsured and working poor, many of whom would otherwise seek primary and routine care in the emergency room [3,8]. The emergence of free clinics is not entirely understood. Literature suggests it is in response to the limitations in the healthcare safety net, coupled with an intransigent uninsured problem [9]. Often, the beginning of a free clinic is described as a convergence of factors: the presence of unmet needs in the community and the willingness of physicians, nurses, and other healthcare professionals to respond to those needs [8-9].

Despite operating through various grants and private donations, free clinics rely heavily on volunteers to ensure their doors stay open. Free clinics range from offering only a few services and are not well-equipped to large-scale operations offering more than the average doctor’s office or community hospital. Free clinics can decrease the number of patients who otherwise use emergency departments as their only source of primary health care and by doing so save hospitals huge amounts of money spent on unnecessary hospital visits [8-9]. Doctor’s offices and urgent cares also benefit from free clinic services for patients who might otherwise end up unable to pay for care [3,8,10]. Even though the patient, in this case, was referred to us by a hospital system, he ultimately was able to receive free high-quality follow-up care, management, and prescriptions for wound care and long-term uncontrolled T2DM.

Cellulitis is one of the most common skin infections seen by primary care offices, urgent care, and emergency departments [11]. Cellulitis typically involves the deeper dermis of subcutaneous fat while skin abscesses are a collection of pus within the dermis or subcutaneous space and accompany cellulitis [1]. Cellulitis is most commonly caused by group A beta-hemolytic Streptococcus and less commonly caused by *S. aureus*, which includes methicillin-resistant strains [11]. Since the first reported US *methicillin-resistant S. aureus* outbreaks in the early 1960s, the prevalence of *methicillin-resistant S. aureus* has increased in both the healthcare and community settings [12-13]. Methicillin resistance is mediated by penicillin-binding protein 2a (PBP-2a), which is encoded by the mecA gene. This gene permits the organism to grow and divide in the presence of methicillin and other beta-lactam antibiotics [11].

Diabetes mellitus (DM) is a common and debilitating disease that can affect multiple organ systems, including the skin. Poor glycemic control has been known to be associated with the development of cellulitis. Type 2 diabetes mellitus (T2DM) is characterized by persistent hyperglycemia, insulin resistance, and relative impairment in insulin secretion (beta-cell dysfunction). Insulin resistance and beta-cell dysfunction are inherently complex regarding their interrelation in the pathogenesis of DM such as induced hyperglycemic states and increased insulin demand [14]. Patients typically present with a constellation of increased fatty acid levels and inflammatory cytokines from fat and oxidative factors [5]. Glycated hemoglobin (HbA1c) is a biochemical marker that is used as the gold standard point-of-care test to estimate the change in glucose values over a three-month period and routinely monitor long-term glycemic control in people with DM.

The delay in diagnosis of this patient’s T2DM could perhaps have further complicated his condition. The pathophysiology of diabetes uncovers ideas that are worth considering when assessing the patient’s healing process. Hyperglycemia is associated with stiffer blood vessels, which cause slower circulation, reducing tissue oxygenation [15]. This is one of a myriad of reasons why the barrier function of the skin is impaired and overall skin integrity is compromised. The patient’s HbA1c dropped to 6.4% 60 days after initiating care with us, which coincided with an improvement in the overall depth of his wound.

## Conclusions

This was a case of an uninsured patient with cellulitis and a deep back abscess complicated by a new diagnosis of poorly controlled type 2 diabetes mellitus who was referred to the Gary Burnstein Clinic for wound management by a nearby hospital. Our discussion serves to showcase the capabilities of free clinics in our community. Not only can these facilities address the needs of vulnerable populations but they can help to decrease the burden caused by individuals overusing emergency departments for acute, chronic, and preventable conditions. The patient also adds to the existing data suggesting that poor glycemic control is associated with cellulitis and further addresses the factors underlying wound management in vulnerable populations.
